# Effects of topoclimatic complexity on the composition of woody plant communities

**DOI:** 10.1093/aobpla/plw049

**Published:** 2016-08-02

**Authors:** Meagan F. Oldfather, Matthew N. Britton, Prahlad D. Papper, Michael J. Koontz, Michelle M. Halbur, Celeste Dodge, Alan L. Flint, Lorriane E. Flint, David D. Ackerly

**Affiliations:** 1 Department of Integrative Biology, University of California, Berkeley, CA 94720, USA; 2 Department of Biological Sciences and Bolus Herbarium, University of Cape Town, Private Bag, Rondebosch 7700, South Africa; 3 Department of Plant Sciences, University of California Davis, Davis, CA 95618, USA; 4 Pepperwood Preserve, 2130 Pepperwood Preserve Road Santa Rosa, CA 95404, USA; 5 Water Resources Discipline, U.S. Geological Survey, Placer Hall, 6000 J Street, Sacramento, CA 95819, USA; 6 Jepson Herbarium, University of California, Berkeley, CA 94720, USA

**Keywords:** California, climatic water deficit, community analyses, oak woodlands, topoclimate, woody vegetation

## Abstract

Topography can create substantial environmental variation at fine spatial scales. Shaped by slope, aspect, hill-position and elevation, topoclimate heterogeneity may increase ecological diversity, and act as a spatial buffer for vegetation responding to climate change. Strong links have been observed between climate heterogeneity and species diversity at broader scales, but the importance of topoclimate for woody vegetation across small spatial extents merits closer examination. We established woody vegetation monitoring plots in mixed evergreen-deciduous woodlands that spanned topoclimate gradients of a topographically heterogeneous landscape in northern California. We investigated the association between the structure of adult and regenerating size classes of woody vegetation and multidimensional topoclimate at a fine scale. We found a significant effect of topoclimate on both single-species distributions and community composition. Effects of topoclimate were evident in the regenerating size class for all dominant species (four *Quercus spp.*, *Umbellularia californica* and *Pseudotsuga menziesii*) but only in two dominant species (*Quercus agrifolia* and *Quercus garryana*) for the adult size class. Adult abundance was correlated with water balance parameters (e.g. climatic water deficit) and recruit abundance was correlated with an interaction between the topoclimate parameters and conspecific adult abundance (likely reflecting local seed dispersal). However, in all cases, the topoclimate signal was weak. The magnitude of environmental variation across our study site may be small relative to the tolerance of long-lived woody species. Dispersal limitations, management practices and patchy disturbance regimes also may interact with topoclimate, weakening its influence on woody vegetation distributions. Our study supports the biological relevance of multidimensional topoclimate for mixed woodland communities, but highlights that this relationship might be mediated by interacting factors at local scales.

## Abstract (precis)

Topographically complex landscapes exhibit large variations in climate. This climate heterogeneity has been linked to high biodiversity and may enable species persistence with a changing climate. It is unclear how woody vegetation composition responds to climate heterogeneity defined by multiple climate variables at topographic scales of 10 -100s of metres. At this scale, we quantified both vegetation composition and climate variables in a topographically complex California woodland and found woody communities to be sensitive to climate variation. However this relationship was weak, implying that local scale ecological processes (*e.g.*, disturbance, dispersal limitation) mediate the effect of topographically driven climate variation.

## Introduction

Woody, canopy-dominant species are crucial, long-lived members of many ecosystems. A wide range of ecological processes determine the landscape patterns of woody vegetation including climate limitations, biotic interactions, priority effects, dispersal and disturbance (Woodward *et al.* 2004; Bond and Keeley 2005). Changes in elevation, slope and aspect create a complex topoclimatic landscape (Ashcroft and Gollan 2013), and these heterogeneous landscapes have been linked to higher ecological diversity at global scales (Kreft and Jetz 2007). Heterogeneous topoclimates can create a patchwork of diverse woody vegetation over short distances and may shape how species respond to changing climate conditions (Whittaker 1967; Dobrowski 2011; Ackerly *et al.* 2015). Thus, the influence of topoclimate on local species distributions is of fundamental importance in both basic (MacArthur 1972) and applied ecology (Lawler *et al.* 2015). Quantification of topoclimate and species diversity at matching scales is a critical first step to understanding the relationship between topoclimate heterogeneity and woody community composition over small spatial extents.

Combinations of topographic features create climatic gradients on the scale of 10s–100s of meters (Geiger *et al.* 2009). Topoclimate is distinguished here from microclimate, which refers to spatial variations in environmental conditions due to vegetation cover or surface features smaller than 10 m (De Frenne *et al.* 2013). Across large changes in elevation, lower elevation sites have warmer overall temperatures, as well as higher variation in daily temperature and lower variation in seasonal temperatures (Korner 2003). However, at the topoclimate scale, this pattern can reverse. Lower elevation sites often have cooler minimum temperatures due to cold-air pooling in valleys. Cold-air pooling in steep-sided valleys and basins can greatly lower night-time temperatures, especially in still air and clear sky conditions (Lundquist *et al.* 2008; Daly *et al.* 2009).

Slope and aspect influence solar radiation exposure, soil properties and disturbance regimes. Equatorial-facing slopes have increased exposure to solar radiation, which increases light availability and maximum daily temperatures relative to polar-facing slopes (hereafter referred to as south- and north-facing, respectively, as this study was conducted in the Northern Hemisphere) (Holland and Steyn 1975). Southwest-facing slopes generally have higher effective heat loading than southeast-facing slopes, despite similar radiation loads, due to higher afternoon temperatures (McCune and Keon 2002). Steeply sloped areas also have reduced soil depth and greater rates of disturbance-induced erosion (Heyerdahl *et al.* 2001; Roering and Gerber 2005). These individual features—elevation, hillslope position, slope and aspect—interact with each other and the regional climate to create complex topoclimate gradients within local landscapes. For instance, increasing slope magnifies the effect of aspect, and increases hill-shading in nearby areas (Flint and Childs 1987).

Topography can also shape topoclimate through local hydrologic processes (Anderson and Kneale 1982). Water runs downhill, evaporates more readily at higher temperature and is less available in the thin soils of steep slopes (Tani 1997; Flint *et al.* 2013). Measurements of a site’s topoclimate should, therefore, incorporate the intensity of solar radiation and availability of soil moisture, as well as their interaction (Stephenson *et al.* 1990). Water balance variables capture the relationships between these components, including their seasonal availability (Stephenson *et al.* 1990). Advances in modeling allow estimation of the following water balance variables at the topoclimatic scale: potential evapotranspiration (PET, mm), actual evapotranspiration (AET, mm) and climatic water deficit (CWD, mm) (Flint *et al.* 2013). CWD is an integrative measure of the cumulative excess of PET relative to AET during the dry season (i.e. when energy availability exceeds water supply), such that CWD = PET−AET.

Topoclimate components, considered separately, have well-documented correlations with species distributions. For instance, belts of vegetation occur along elevation contours (Whittaker and Niering 1965), and aspect has variable effects on species diversity and community composition (Armesto and Martinez 1978; Weiss *et al.* 1988; Sage and Sage 2002; Bennie *et al.* 2006; Harrison *et al.* 2010). Furthermore, integrated measures of both temperature and soil moisture in heterogeneous landscapes are strong drivers of vegetation distributions (Stephenson 2015). CWD is a particularly good predictor of woody vegetation distributions, as well as temporal woody vegetation change, in semi-arid landscapes (Lutz *et al.* 2010; Crimmins *et al.* 2011; Das *et al.* 2013; Stephenson 2015). Increasing CWD has been linked to changes in tree recruitment, growth and mortality, as well as community composition (Salzer *et al.* 2009; Millar *et al.* 2015). Reductions in large tree densities and shifts toward more oak-dominated landscapes in the last century in California have been strongly correlated with increasing CWD (McIntyre *et al.* 2015). However, it remains unclear the extent to which variation in multiple topoclimate dimensions, considered in concert, can explain woody vegetation diversity at the local scale (but see Van de Ven *et al.* 2007).

Landscapes with heterogeneous topoclimates have been championed as valuable conservation units for protecting both current and future biodiversity (Ackerly *et al.* 2010; Lenoir *et al.* 2013; Lawler *et al.* 2015). In the context of rapid climate change, landscape heterogeneity reduces the rate at which a species must move to track its climate niche and increases the availability of cooler, wetter refugia (Loarie *et al.* 2009; Dobrowski 2011). Heterogeneous landscapes harboring high levels of biodiversity may also provide thermophilic propagules for community reassembly (Ackerly *et al.* 2010). Most protected areas in North America have a small spatial extent, and land management and acquisition decisions take place at this scale (Chape *et al.* 2005; Heller and Zavaleta 2009). Thus further research on vegetation–climate relationships at a local scale is a conversation priority, especially in the face of 21st century climate change.

We quantified woody community diversity and topoclimate complexity at a matching local scale in mixed evergreen-deciduous woodlands of Northern California. We established woody vegetation survey plots that span a wide range of topoclimate variability across a local landscape. Woody vegetation may exhibit size-dependent sensitivity to topoclimate (Máliš *et al.* 2016) and regenerating individuals may require a different suite of climatic conditions to establish than adults require to persist (Grubb 1977; Jackson *et al.* 2009; Mclaughlin and Zavaleta 2012; Millar *et al.* 2015). Thus, both adult and regenerating size classes were surveyed and their relationships to topoclimate were analyzed separately.

We asked the following questions: (1) Which components of topoclimate influence local species distributions? (2) Across the species, do the adult and regeneration size classes exhibit different responses to topoclimate gradients? (3) To what extent does topoclimate heterogeneity explain variation in community composition and is this relationship similar for both size classes?

## Methods

### Study site and plot establishment

This study was conducted across the 1263 ha Pepperwood Preserve in northern California (Sonoma Co., 38.57°N, −122.68°W). The preserve is topographically heterogeneous and features vegetation representative of California Coast Ranges, including chaparral, grasslands, Douglas-fir forest, oak woodland and mixed hardwood forest (Halbur *et al.* 2013). Pepperwood is in a transition zone between southern and central California woodlands, dominated by *Quercus agrifolia* along the coast and *Q. douglasii* inland, and northern woodlands with high abundance of *Pseudotsuga menziesii* and *Q. garryana* (a close relative of *Q. douglasii*). There is an extensive land-use history at this preserve, including logging, charcoal making and livestock grazing from the 1800s to the present (Evett *et al.* 2013). There were two large fires on the preserve in 1964 and 1965, and no large fires have occurred since that time (Halbur *et al.* 2013).

Fifty 20 × 20 m woody vegetation-monitoring plots (2 ha in total) were established across Pepperwood Preserve (Ackerly *et al.* 2013). The plot locations were selected based on two criteria: (1) stratification across the topographic gradients of the preserve, and (2) a balanced spread across deciduous and evergreen woodlands, based on a recently completed vegetation map of the preserve (Halbur *et al.* 2013). The following topographic variables were used to stratify the plot locations: elevation, slope, aspect, modeled March radiation, topographic position index (TPI), percent lower pixels (PLP) and topographic water index (TOPOID) (see definitions below).

**Figure 1.**
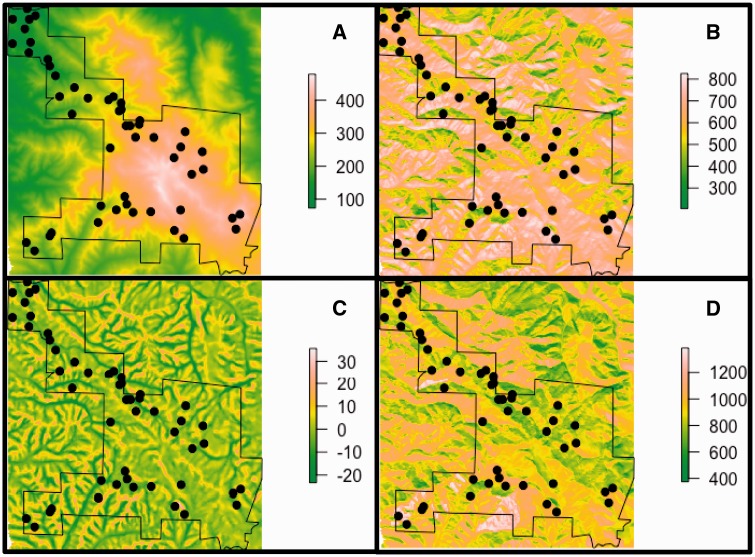
Maps of plots (black circles) across Pepperwood Preserve (black outline) with the following base layers: (A) DEM (m), (B) March Radiation (kWh/m^2^), (C) TPI (m) and (D) CWD (mm).

Topographical variables were obtained with GIS analyses of a 10-m digital elevation model for Pepperwood Preserve (Fig. 1). Slope and aspect were calculated using the *terrain* function in the *raster* package in *R* (Hijmans *et al.* 2015). Average incident solar radiation (kW h/m^2^) was calculated for each month, based on slope, aspect and local topographic shading, in the Solar Analyst function of the Spatial Analyst library in ArcGIS (Fu and Rich 2000). March radiation was used for plot selection and for analyses reported here because it represents radiation during the spring growing season. TPI and PLP offered alternative metrics of local topographic relief. TPI (m) is the elevation of a pixel minus the mean elevation of the landscape in a defined radius (positive values indicate upper slope and hilltop positions, negative values are lower slope or valley bottoms) (Weiss 2001). PLP (%) is calculated as the percent of pixels within a specified radius that are lower than the focal pixel (ranges from 0 to 100, with higher values for upper slope positions). Both these topographic relief metrics were calculated with a neighborhood radius of 100 m. The metrics were similar with a neighborhood radius of 500 m, so we only used calculations derived from the 100 m radius. TOPOID was calculated using a hydrologic flow accumulation algorithm that incorporates the amount of ‘upstream’ area from which water would flow towards a focal pixel and the slope of that area (flatter locations with more upstream area will have a greater TOPOID value).

**Figure 2.**
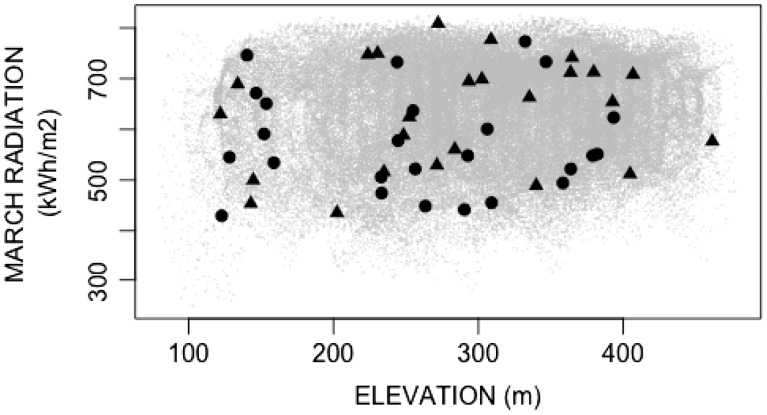
The plot locations span a large amount of the climate space of the preserve. The gray dots represent all combinations of elevation and average March radiation across Pepperwood Preserve. The shapes represent different vegetation types of the plots, with triangles = evergreen woodland, and circles = deciduous woodland. These two climate variables are representative of the two main principal components, illustrating that this figure is representative of many of the combinations of the climate parameters measured across the preserve.

Across the plots, elevation ranged from 122 m to 462 m, average March radiation ranged from 430 kW h/m^2^ to 809 kW h/m^2^, TPI ranged from −10 m to 14 m, PLP ranged from 14 % to 99 % and TOPOID ranged from 3.5 to 9.6. As plots were installed, we reexamined their distribution across topographic gradients and vegetation types, adding new locations to fill gaps until we achieved a well-stratified distribution across the entire preserve (Fig. 2). Each plot was placed on a homogeneous slope and aspect so that orientation could be clearly characterized, resulting in a bias away from sampling on ridge tops, valley bottoms or strongly curved hill slopes.

### Vegetation monitoring

For this study, we included all species with a woody growth form represented by at least one individual with a greater than 1 cm diameter at breast height (DBH) in at least one plot across the study (Table 1). DBH was measured at 1.4 m. Poison oak (*Toxicodendron diversilobum*) was abundant but not sampled, as it is generally below the threshold size and presents a health hazard. Each 20 × 20 m plot was subdivided into sixteen 5 × 5 m quadrats for vegetation sampling, but all data are reported at the plot level. All individuals of the study species were sampled in the 50 plots and categorized into one of the five size classes: seedling, juvenile, sapling, tree and stump sprout. Seedlings were defined as individuals <10 cm height. Juveniles were defined as individuals with a height of ≥ 10 and < 50 cm. Saplings were defined as individuals with a height ≥ 50 cm and DBH of < 1 cm. Trees were defined as individuals with a DBH of ≥ 1 cm. Stump sprouts were defined as individuals with the same specification as a sapling, but observed to be growing from a larger tree or stump. All individuals of the five size classes were identified to species. Saplings, trees and stump sprouts were tagged with uniquely numbered metal tags, and locations recorded to the nearest cm, relative to the corner of each quadrat. For saplings and stump sprouts, we measured the height of the individual, the basal diameter of the main (largest basal area) stem at 10 cm off the ground, and the number of stems that split below 10 cm. Basal area was calculated for trees based on DBH. Seedlings and juveniles of each species were censused in each plot. These methods allow comparison with other standardized woody vegetation monitoring protocols (Condit 1998; Gilbert *et al.* 2010). Plot establishment and baseline data collection for tagged individuals occurred in spring 2013. Abundance of seedlings and juveniles were resurveyed in spring 2015 to confirm identifications, and we used the 2015 seedling and juvenile data in this analysis.

**Table 1:** The woody species range in overall abundance for the different size classes. The following metrics are cumulative across all plots: basal area = tree basal area (cm^2^), TR = number of individuals in the tree size class, SA = number of individuals in the sapling size class, JU = number of individuals in the juvenile size class, SE = number of individuals in the seedling size class.

Code	Common name	Botanical Latin	Basal area	TR	SA	JU	SE
ACEMAC	Bigleaf Maple	*Acer macrophyllum*	0	0	0	10	6
ADEFAS	Chamise	*Adenostoma fasciculatum*	16	2	3	0	0
AESCAL	Buckeye	*Aesculus californica*	3084	9	5	53	0
AMOCAL	Napa false indigo	*Amorpha californica* var. *napensis*	255	31	242	13	0
ARBMEN	Madrone	*Arbutus menziesii*	31 931	66	101	246	230
ARCMAN	Manzanita	*Arctostaphylos* sp.	4360	15	7	4	0
BACPIL	Coyote Bush	*Baccharis pilularis*	21	1	11	2	0
CEACUN	Wedgeleaf Ceanothus	*Ceanothus* cuneatus	0	0	23	0	0
CERBET	Mountain Mahogany	*Cercocarpus betuloides*	0	0	5	20	1
FRACAL	Coffee berry	*Frangula californica*	46	10	103	183	69
HETARB	Toyon	*Heteromeles arbutifolia*	3393	159	156	99	11
NOTDEN	Tan oak	*Notholithocarpus densiflorus*	564	5	7	12	1
PSEMEN	Douglas-fir	*Pseudotsuga menziesii*	173 320	441	437	800	977
QUEAGR	Coast live oak	*Quercus agrifolia*	208 574	244	273	762	191
QUEBER	Scrub oak	*Quercus berberidifolia*	22	6	31	33	6
QUEDOU	Blue oak	*Quercus douglasii*	55 385	68	0	1561	630
QUEGAR	Oregon oak	*Quercus garryana*	170 152	301	5	2247	606
QUEKEL	Black oak	*Quercus kelloggii*	47 375	58	1	184	48
QUELOB	Valley oak	*Quercus lobata*	1320	1	0	2	0
QUEWIS	Interior live oak	*Quercus wislizenii*	31	1	0	0	0
TORCAL	California nutmeg	*Torreya californica*	0	0	3	3	0
UMBCAL	California bay laurel	*Umbellularia californica*	19 546	168	889	2060	163

### Environmental measurements

Beginning in 2013, temperature and relative humidity were monitored in all plots at 30-minute intervals. A HOBO datalogger (Hobo model U23, Onset Corp., Bourne, MA) nested inside a solar radiation shield was placed at 1.2 m above the ground, and 5 m outside the plot edge in a location of similar light availability and species composition. Annual winter minimum temperature was calculated as the average daily minimum temperature for the months of November and December in 2014, and ranged from 6.4 °C to 10.5 °C across the plots. Annual summer maximum temperature was calculated as the average daily maximum temperature for the months of July and August in 2014, and ranged from 26.0 °C to 32.6 °C across the plots. Soil moisture measurements were taken as volumetric water content in the center of each quadrat in every plot at a depth of 12 cm (Campbell Hydrosense, Model CS659). The mean of all measurements across the 16 quadrats was calculated to determine the average soil moisture of each plot. These readings were taken for the 2013–2015 field seasons in all plots in the first week of May within a 5-day window without precipitation. Thus, measurements were taken prior to the onset of summer, when soils may become uniformly dry. The average soil moisture ranged from 2.6 % to 14.5 % across the plots.

Water balance parameters for the plots were obtained from a 10 m resolution downscaled analysis of the Basin Characterization Model (Flint et. al. 2013) (Fig. 1D). The Basin Characterization Model is a water balance model that takes into account soils, precipitation, hydrology and temperature to model spatial patterns of AET, PET and CWD. Gridded data were obtained from PRISM and downscaled using the Gradient-Inverse-Distance-Squared algorithm based on the 10 m digital elevation model of the local landscape. Monthly modeled values of AET, PET and CWD were summed to obtain water year totals, and a 30-year (1981–2010) average was used for the analyses in this paper. The 30-year average of each of the modeled water balance parameters were averaged across the four 10 m grid cells within each plot to obtain a single plot value. Across the plots, AET ranged from 128 mm to 455 mm, PET ranged from 878 mm to 1516 mm and CWD ranged from 654 mm to 1314 mm.

### Statistical analyses

A principal component analysis (PCA) was performed with *princomp* on 11 of the environmental variables quantified across the plots to reduce the dimensionality, but to still include the contributions of all variables to overall patterns of topographic heterogeneity (Oksanen *et al.* 2008). The PCA included modeled parameters (AET, PET, CWD, elevation, March radiation, TOPOID, TPI and PLP) and field-measured parameters (2014 winter minimum temperature, 2014 summer maximum temperature and 2014 soil moisture). All climate variables were scaled and centered prior to the PCA. The first two principal components, PC1 and PC2, of this analysis were used as independent variables for subsequent analyses of vegetation distributions. The correlations between these two principal components and the topoclimate variables of interest were quantified with the *dimdesc* function in R (Husson *et al.* 2015).

Using the first two topoclimate principal component scores, we asked which topoclimate variables affected single-species distributions (Question 1) and whether this relationship depended on size class (Question 2). We considered two size classes: all trees were considered as the “adult” size class and all seedlings and juveniles were considered together as the “regeneration” size class. For these first two questions, we focused on the six most dominant woody species across the plots: *Q. douglasii*, *Q. garryana*, *Q. agrifolia*, *Q. kellogii*, *P. menziesii* and *Umbellularia californica*. These six species each have greater than 50 % basal area in at least one plot, and together account for 71 % of all basal area across the study. For each species, the adult size class and the regeneration size class were analyzed separately and then compared. Linear regressions were used to assess the relationship between adult abundance (total basal area per plot), and the two topoclimate principal components (PC1 and PC2). Binomial regressions were used to assess the probability of presence of individuals in the adult or regeneration size classes across PC1 and PC2. When analyzing the regeneration size class, we also included an additional covariate of conspecific adult basal area as a proxy for local seed rain. Poisson regression was used to examine the sensitivity of regeneration abundance to PC1, PC2 and conspecific basal area. Conspecific basal area was included in the regeneration models as a main effect and as an interaction term with PC1 and PC2. All covariates were scaled and centered, and the model with the best fit, based on AIC, was selected for each species.

We separately assessed the effect of topoclimate on community composition for the adult and regeneration size classes (Question 3) using conditional, constrained redundancy analyses (CCRA). The constrained redundancy analysis is a form of multivariate regression in which the response variable is the community dissimilarity across plots (Anderson *et al.* 2011). The conditional version of the constrained redundancy analysis removed the effect of evergreen versus deciduous physiognomy on community dissimilarity (Legendre 2007). This conditioning was necessary because a portion of the community dissimilarity arose from our non-random plot placement, which intentionally represented both evergreen and deciduous vegetation types across the topoclimate gradients (Fig. 2). We used the square-root of the Bray–Curtis dissimilarity metric as the response variable in the CCRA with PC1, PC2 and spatial distance between plots as covariates (Anderson *et al.* 2011). Mantel tests showed spatial autocorrelation in PC1, PC2 and the community matrices which motivated us to include spatial distance between plots as a covariate (Mantel and Valand 1970; Legendre 1993) (**see Supporting Information – Table S2**). The spatial distance between plots was represented in the CCRA by the first principal component of the spatial distance matrix. From the R *vegan* package, we used the *capscale* function to perform the CCRA, and the *adonis* function to determine the variation explained by each significant model parameter (McArdle and Anderson 2001; Oksanen *et al.* 2008). Lastly, we used PC1 and PC2 to predict adult and regeneration species richness in a multiple regression framework.

**Table 2:** For most species, there is significant three-way interaction between the topoclimate axes and conspecific adult basal area for models of the regeneration abundance. PC1, first axes of the topoclimate PCA; PC2, second axes of the topoclimate PCA; TR, conspecific adult basal area. ‘:’ indicates an interaction between the parameters. ‘+’ indicates a significant positive effect of that parameter, and ‘−’ indicates a significant negative effect. *G*^2^, the difference in deviance explained by the null model (null deviance) and full model (residual deviance). Wald tests between the null and full models are all highly significant for all species. DF, degrees of freedom.

Species	TR	PC1	PC2	PC1:PC2	PC1: TR	PC2: TR	PC1: PC2: TR	Null deviance; DF = 49	Residual deviance; DF = 42	*G*^2^
*Quercus agrifolia*	+				−	+	−	2154	637	1517
*Quercus garryana*	+	+	+		+	+	−	8076	4643	3424
*Quercus douglasii*	+	+	+	−	+	−		7787	2368	5149
*Quercus kellogii*	+		+	−	+		+	865	340	525
*Pseudotsuga menziesii*	+	+	−	+	−	+	−	6937	4091	2846
*Umbellularia californica*	+	–	–	+	+		+	3880	2207	1673

## Results

**Figure 3.**
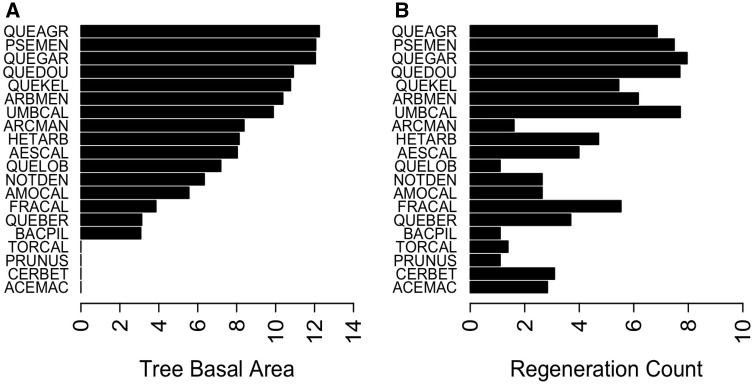
(A) Tree abundance in descending order, based on basal area (cm^2^). (B) Regeneration abundance, based on counts. For species names, see Table 1. Both abundance metrics are log-transformed.

Across the 50 plots, a total of 3,900 individuals were tagged and mapped (2312 saplings and 1588 trees), and 11 235 additional individuals ≤50 cm tall were recorded and enumerated by quadrat, but not tagged (2939 seedlings and 8296 juveniles). Twenty-five species were identified and species richness within a plot ranged from 3 to 13 species (Table 1). Tree densities ranged from 3 to 208 individuals per plot. Total basal area (the sum of trunk cross-sectional areas) for trees ranged from 10.8 to 94.8 m^2^ ha^−^^1^. Across all plots, the number of seedlings and juveniles per plot ranged from 14 to 868. The most abundant tree species based on the number of individuals was *P. menziesii*, followed by *Q. garryana*, *Q. agrifolia* and *U. californica*. However, the most abundant tree species based on the basal area was *Q. agrifolia*, followed by *P. menziesii* and *Q. garryana* (Fig. 3). The most common species of the regeneration size class (seedling + juvenile counts) in declining order were *Q. garryana*, *U. californica*, *Q. douglasii* and *P. menziesii* (Fig. 3).

### Topoclimate principal components

**Figure 4.**
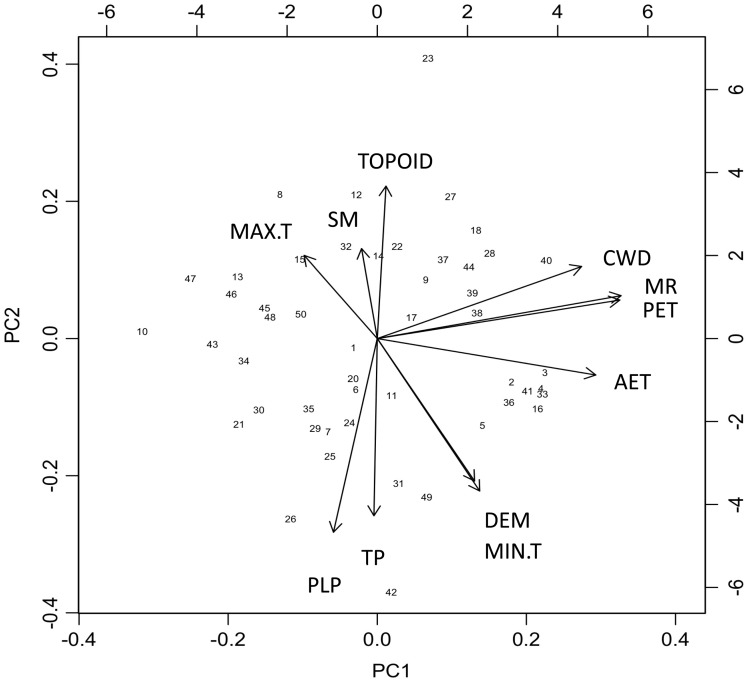
Biplot of the principal components analysis for the 11 environmental variables quantified across the plots. CWD, climatic water deficit (mm); AET, actual evapotranspiration (mm); PET, potential evapotranspiration (mm); MR, March radiation (kWh/m^2^); DEM, elevation (m); MIN.T, 2014 annual minimum winter temperature (°C); MAX.T, 2014 annual maximum summer temperature (°C); SM, 2014 soil moisture measurements (%); TPI, topographic position index (m); PLP, percent lower pixels (%); TOPOID, topographic water index.

PC1 and PC2 explained 59 % (33 % and 26 %, respectively) of the environmental variation observed across plots (Fig. 4). PC1 was significantly positively correlated with CWD (*R*^2 ^=^ ^80 %), AET (*R*^2^=85 %), PET (*R*^2 ^=^ ^95 %) and March radiation (*R*^2^=95 %). PC2 was significantly positively correlated with maximum summer temperature (*R*^2^=35 %), soil moisture (*R*^2^=38 %) and TOPOID (*R*^2^=65 %), and negatively correlated with elevation (*R*^2 ^=^ ^61 %), minimum winter temperature (*R*^2 ^=^ ^65 %), TPI (*R*^2 ^=^ ^76 %) and PLP (*R*^2 ^=^ ^81 %).

### Adult responses to topoclimate

Only three of the six dominant woody species distributions showed significant relationships with the preserve’s topoclimate for the adult size class. Both *Q. garryana* and *Q. agrifolia* adult abundance were correlated with PC1. *Q.* *garryana* had a negative relationship with PC1, with 8 % of the variation in its abundance explained (*P* = 0.016). *Q.* *agrifolia* had a positive relationship with PC1, with 15 % of the variation in its abundance explained (*P* = 0.002). The effect of PC1 and PC2 on the probability of the presence of these two species showed a similar pattern. *Umbellularia* *californica* abundance was not significantly explained by either PC1 or PC2, however, *U. californica* presence had a weakly significant negative relationship with PC1 (*P* = 0.048). For the adult size class, none of the dominant species showed any relationship with PC2 for either abundance or presence.

### Regeneration responses to topoclimate

**Figure 5.**
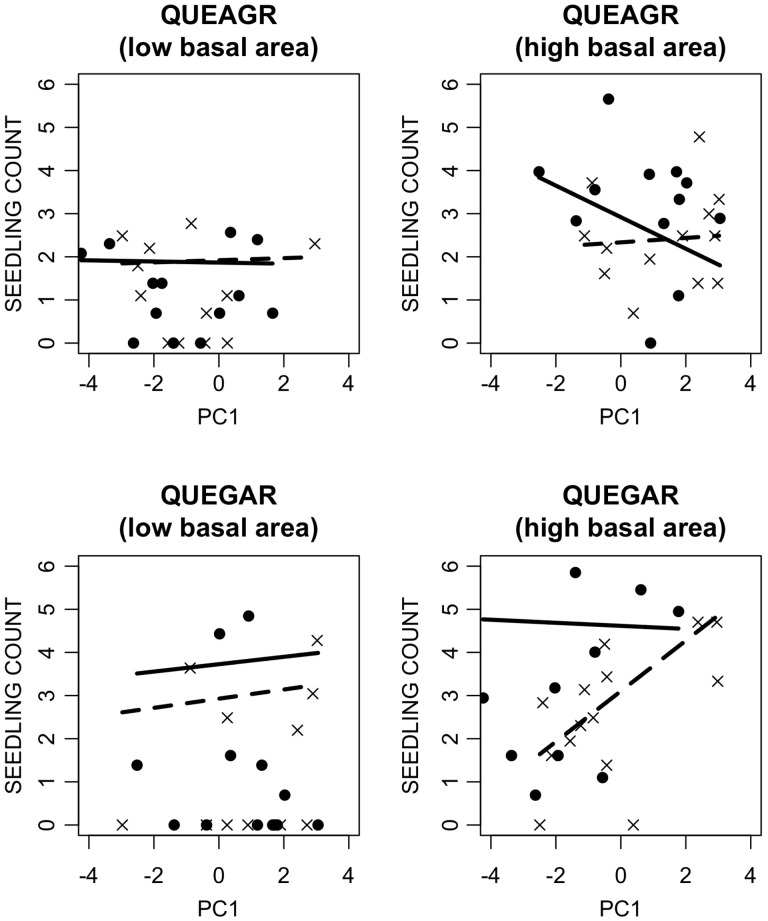
Model predictions show interactions among PC1, PC2, and adult abundance (basal area) for natural log-transformed regeneration abundance of *Q. agrifolia* (QUEAGR) and *Q. garryana* (QUEGAR). Dashed lines are model prediction from the lower 50th percentile PC2 values, and solid lines are from the upper 50th percentile PC1 values. The *x*’s represent data from plots in the lower 50th percentile for PC2 values and points represent data from plots in the upper 50th percentile for PC2 values. The figures on the left are predictions for the lower 50th percentile of conspecific adult basal area and the figures on the right are predictions for the upper 50th percentile of conspecific adult basal area.

The best model fits for regeneration presence/absence included only conspecific adult abundances for all six dominant species. Thus, the effect sizes of PC1 and PC2 on the probability of species presence were not further analyzed. The best model fits for regeneration abundance included PC1, PC2, conspecific adult abundance and their interactions. Conspecific adult abundance had a positive main effect on regeneration abundance for all dominant species (Table 2). The effect of PC1 on regeneration abundance was significant for most of the dominant species, except *Q. kellogii* and *Q.agrifolia*, and the sign of the effect was predominantly positive (higher abundance on south-facing slopes) (Table 2). For most of the *Quercus* spp., PC2 had a significant positive main effect on regeneration abundance (Table 2). For *U. californica* and *P. menziesii*, PC2 had a significant negative effect of regeneration abundance (higher abundance on upper hill slopes) (Table 2). The abundance of conspecific adults also influenced the effect of PC1 and PC2 on regeneration abundance via their interactions (Fig. 5). Many two and three-way interactions between PC1, PC2 and adult conspecific basal area were significant (Table 2 and Fig. 5). However, no obvious patterns of these effects emerged across species.

### Community responses to topoclimate

**Figure 6.**
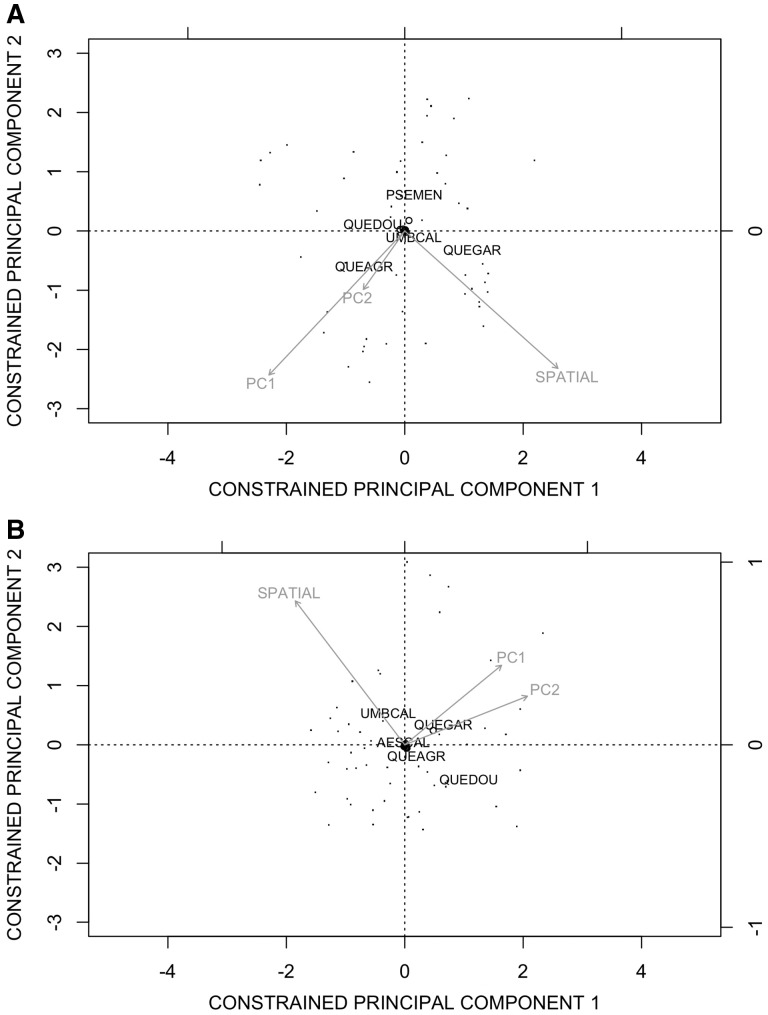
The first two principal components of the constrained redundancy analyses on the adult community (A) and the regeneration community (B). The points represent the 50 sites. Each arrow represents the direction and magnitude, indicated by length, of the effect of the topoclimate axes (PC1 and PC2), and the distance between plots (SPATIAL). The species code locations represent how the species are structured in the constrained ordination space. For species names see Table 1.

A small amount of variation in both adult and regeneration community composition was explained by PC1, PC2 and the spatial distance between plots. The adult community structure across the preserve was significantly correlated with PC1 (10 % variation explained, *P* = 0.007) and spatial distance between plots (9 % variation explained, *P* = 0.001) (Fig. 6A). The regeneration community structure was significantly correlated with PC2 (5 % variation explained, *P* = 0.013) spatial distance (4 % variation explained, *P* = 0.009) and PC1 (3 % variation explained, *P* = 0.041) (Fig. 6B). Lastly, adult species richness was negatively correlated with PC1 (10 % variation explained, *P* = 0.009), but had no relationship with PC2. Species richness of the regeneration size class was not correlated with either PC1 or PC2.

## Discussion

By examining the distribution of adult and regeneration size classes of woody vegetation across 50 plots that span the climate space of a single preserve, we assessed whether topoclimate heterogeneity was ecologically relevant for woody plant species distributions and community composition at this scale. Overall, we found support for species distributions and community composition being, in part, influenced by the topoclimate variation on the landscape. Although statistically significant, the effects of topoclimate on single-species distributions, community composition and species richness were small. Here, we discuss the observed vegetation patterns related to topoclimate and suggest biological mechanisms that may contribute to the relatively small size of the effects in this study.

Our study adds to a relative paucity of woody vegetation studies in Mediterranean climates with mapped stems including small individuals in regeneration size classes (Gilbert *et al.* 2010; Anderson-Teixeira *et al.* 2015). These vegetation plots were dominated by *Quercus* spp. with high oak basal area, species diversity, and a mix of both evergreen and deciduous oaks. Relative to the UCSC Forest Ecology Research Plot, a single large woody vegetation plot in a comparable climate, our most dominant species contributed to much less of the total basal area (Gilbert *et al.* 2010). This difference perhaps reflects that our sampling strategy encompassed multiple vegetation types. By sampling 2 ha of forest across 50 plots, we were able to capture variation in size structure, abundance and composition in the woody vegetation across the 1263 ha preserve.

The topoclimate principal components (PC1 and PC2) represented combinations of topographic features observed across the preserve. At this local scale, all the PC1 parameters (March radiation, PET, AET and CWD) were highly positively correlated, likely due to strong effects of slope and aspect on solar radiation load. Positive PC1 values were associated with south-facing slopes where the radiation load is higher. Low elevation valleys were associated with positive PC2 values, with wetter soils and cooler temperature minimums due to nighttime cold-air pooling. Negative PC2 values were associated with drier, high-elevation ridges and upper hill slopes.

Adults showed species-specific responses to the environmental parameters of PC1. This suggests that the role of water availability and temperature for woody vegetation abundance at the topoclimate scale is primarily reflected in the interaction and seasonality of these climate variables (Stephenson *et al.* 1990). The two dominant oak species, *Q. garryana* and *Q. agrifolia*, showed opposite responses in abundance to PC1. The deciduous *Q.* *garryana* was more abundant in sites with less solar radiation and lower CWD. The evergreen *Q. agrifolia* was more abundant on sites with higher AET, and occupied sites with the highest CWD. These opposing patterns may reflect differences in these species’ ranges; Pepperwood Preserve is near the southern range limit of *Q. garryana*, and the northern range limit of *Q. agrifolia*. These species may be found in topoclimates that exhibit environmental conditions more similar to those found in their range centers (Holland and Steyn 1975). However, geographic range limits may not necessarily be at the edges of the climatic niche of the species (Chardon *et al.* 2015). Further research is needed to assess the degree to which range-wide climate characteristics influence local-scale distributions for this system.

None of the dominant woody species were correlated with the topoclimate principal component associated with cooler, wetter valley bottoms (PC2) for the adult size class. It is possible that the range of climate variation present across the PC2 topoclimate gradient of Pepperwood Preserve is narrow relative to the climate tolerances of these woody species. Although we observed topoclimate sensitivity in the presence and abundance of the adults of some species, the greatest amount of variation explained was only 15 % (*Q. agrifolia*) and three of the six dominant species showed no sensitivity to either topoclimate gradients. The magnitude of topography necessary to cause a response is most likely species specific.

As opposed to the adults, the regeneration size class showed sensitivity to both the topoclimate principal components and the interactions between them. This result aligns with other studies that show that seedlings of woody vegetation may be sensitive to environmental variation at smaller scales than adults (Gray and Spies 1997; Máliš *et al.* 2016). Despite some general trends, there is strong evidence for highly species-specific responses in the two-way and three-way interactions between the topoclimate principal components and conspecific adult basal area. Abundance of seedlings and juveniles generally increased with conspecific adult basal area for all species. In some cases, high seed input by abundant conspecific adults can overwhelm the effects of variable conditions and effectively suppress the topoclimate sensitivity of the regeneration size class (Clark *et al.* 1998; Warren *et al.* 2012). However, we were able to detect a signal of both the conspecific adult abundance and environmental parameters on regeneration abundance. Our results support that local (within 20 m) seed source is the main driver of the probability that recruitment is observed at a site, but the overall abundance of the regeneration class is mediated by the topoclimate.

There may be ecological constraints on the capacity of a heterogeneous landscape to buffer vegetation response to climate change. Replacement of adults by new recruits can be slowed if dispersal is limiting (Aitken *et al.* 2008). We found that conspecific adult abundance, a proxy for local seed input, greatly impacts recruitment, potentially demonstrating dispersal limitation effects on woody species composition at a small spatial extent. Species ability to track their climate niche in response to a changing climate may be delayed due to lack of seed input even at the topoclimate scale. Heterogeneous topoclimates may more likely enable species persistence in favorable sites (i.e. refugia) as the overall species range contracts (Ackerly *et al.* 2010; Dobrowski 2011). This result also warrants further investigation into the microclimate effect of canopy cover on the understory environment occupied by seedlings and juveniles (De Frenne *et al.* 2013; Dobrowski *et al.* 2015).

For both size classes, topoclimate explained some variation in community composition. In concordance with the individual species responses, only the principal component associated with the water balance parameters (PC1) was correlated with variation in the adult community composition. Also the regeneration community composition was correlated with both topoclimate principal components. Previous research has shown a sizable effect of topoclimate on the abundance of short-lived plants (herbs) across a landscape (Harrison *et al.* 2010). For long-lived woody vegetation, we found that in total, only 11 % percent of adult community composition and 7 % of the regeneration community composition were explained by the topoclimate across the preserve. It is possible that the low amount of woody community variation explained by topoclimate is due to the limited range of topoclimate variation at Pepperwood, relative to the environmental tolerances of the studied woody species. We captured a wide range of topoclimate across a small spatial extent, but many of the studied species have broad ranges. Long-lived species, such as woody plants, may also be in disequilibrium with the present climate, impeding our understanding of their climate niche (Svenning and Sandel 2013).

Pepperwood Preserve’s history of fire and land-use may also be limiting the role of topoclimate in shaping woody community distributions. Previous disturbances (natural and anthropogenic) interact with climate to shape vegetation distributions, especially at local scales (Delcourt and Delcourt 1988). Pepperwood Preserve had two major fires in the last 50 years (1964 Hanley Fire and 1965 Calistoga Fire) that burned in approximately two-thirds of our plot locations. Fires will have different effects on species, with some species resprouting after fire (e.g. *Quercus* spp. and *U. californica*) while others needing to re-invade a burned area (e.g. *P. menziesii*, in which all but the largest trees are killed by fire) (Keeley *et al.* 2005). Non-pristine habitats, such as managed farmlands, also may have protracted historical effects on species local distributions. Vegetation in these managed areas may not show sensitivity to topoclimates due to the long-term inertia of vegetation following disturbances (Bodin *et al.* 2013). Evett *et al.* (2013) found increasing tree density, woody encroachment into grasslands and changing community composition (e.g. increased *U. californica*) at Pepperwood Preserve since the early 1900s, and attributed these shifts to changes in land-use and management.

At small spatial extents, ecological processes other than climate limitations may play a prominent role in shaping vegetation communities. Local dispersal limitation may prevent a species from establishing in a suitable topoclimate (Verheyen *et al.* 2003). The stochastic nature of colonization and historical contingencies may also moderate the community dynamics of a site (Duarte *et al.* 2006; Walker and Wardle 2014). Spatial distance between plots, even at this small spatial extent, explains some community variation for both size classes. This pattern may be driven by spatially aggregated land use or disturbances (e.g. fire history), limited seed dispersal at a scale smaller than the preserve or climate patterns at the preserve scale not directly associated with topography (e.g. fog input, as Pepperwood sits at the edge of the Pacific fog belt) (Torregrosa *et al.* 2016).

Future work on the role of topoclimate for woody vegetation distributions and dynamics in this system will focus on range-wide climatic characteristics and functional traits of the species, rather than the species identity per se. This method may reduce the confounding effect of historical contingencies and identify patterns of functional redundancy, in which a topoclimate is equally suitable for different species. Previous work with this trait-based approach has been beneficial in understanding vegetation–climate relationships at the landscape scale (Lenoir *et al.* 2013; De Frenne *et al.* 2013). Comparing results of analyses at the species versus functional level will demonstrate the contribution of topoclimates to the maintenance of both functional and species diversity.

## Conclusion

Downscaled climate variables paired with fine scale vegetation data represent a unique opportunity to resolve fundamental ecological questions regarding the maintenance of species distributions and community types across local topography. There has been a resurgence of interest in this question due to the potential role of topography in how species respond to climate change (Rapacciuolo *et al.* 2014), and the potential importance of small-scale topography in future conservation strategies (Lawler *et al.* 2015). To protect our forests, we need to have a better understanding of topography–vegetation relationships in local landscapes with past disturbances (Millar and Stephenson 2015). We show that disturbed (both naturally and through management) lands can capture community diversity with topographic complexity. However, wide climate tolerances of species and historical contingencies may weaken the relationship between topoclimate and woody vegetation. Our study not only addresses impacts of topography on woody vegetation on small spatial extents but also serves as a baseline for long-term studies of vegetation dynamics in response to climate change in heterogeneous landscapes.

## Sources of Funding

Our work was funded by the Gordon and Betty Moore Foundation (California, USA) Grant nos. 4430 and 2861. Additional support was provided by the US National Science Foundation Graduate Research Fellowship Grant DGE-1106400 (to M.F.O.) and Graduate Research Fellowship Grant DGE-1321845 Amend. 3 (to M.J.K.).

## Contributions by the Authors

D.D.A., M.F.O., M.N.B. and M.M.H. conceived and designed the research. M.F.O., M.N.B., P.D.P., M.J.K., M.M.H., C.D., A.L.F. and L.E.F. performed the research. M.F.O. analyzed the data. M.F.O., M.J.K. and D.D.A. wrote the paper.

## Conflicts of Interest Statement 

None declared.

## Supplementary Material

Supplementary Data

## Supporting Information

The following additional information is available in the online version of this article —

**File 1. Table.** Lists the coordinates, field-measured environmental variables, vegetation statistics, and modeled topographic variables for all 50 plots. UTM.N = Northing coordinate, UTM.E = Easting coordinate, MIN.T = minimum winter temperatures (°C), MAX.T = maximum summer temperatures (°C), SM = 2014 average volumetric water content (%), DEC = percentage of deciduous individuals, DIV = species richness, ELE = elevation (m), MR = March Radiation (kWh/m^2^), TOPOID = topographical water index, TPI = topographic position index (m), PLP = percent lower pixels (%), AET = actual evapotranspiration (mm), PET = potential evapotranspiration (mm), CWD = climatic water deficit (mm); the last three are derived from analysis of the Basin Characterization Model on a 10 m digital elevation model (Flint *et al.* 2013).

**File 2. Figure.** Regeneration model predictions for the additional dominant woody species (*Q. douglasii*, *Q. kellogii*, *P. menziesii*, and *U. californica*). All counts of regeneration abundance are log-transformed. Dashed lines are model prediction from the lower 50^th^ percentile PC2 values, and solid lines are from the upper 50^th^ percentile PC1 values. The x’s represent data from plots in the lower 50^th^ percentile for PC2 values and points represent data from plots in the upper 50^th^ percentile for PC2 values. The figures on the left are predictions for the lower 50^th^ percentile of conspecific adult basal area and the figures on the right are predictions for the upper 50^th^ percentile of conspecific adult basal area.

**File 3. Table.** Mantel test results based on Pearson's product-moment correlation with 1e + 05 permutations. These tests measure the correlation between the spatial distance between plots (SPATIAL) and the dissimilarity of the topoclimate principal components (PC1, PC2), and the correlation between SPATIAL and the vegetation community Bray-Curtis dissimilarity for both adult basal area (ADULT) and regeneration counts (REGEN).
