# Supplementary material for: Effects of topoclimatic complexity on the composition of woody plant communities
Source: AoB Plants. 2016 Aug 2;8:plw049. doi: 10.1093/aobpla/plw049 (PMC4972463; doi:10.1093/aobpla/plw049)
Supplement: Supplementary Data [file supp_8_plw049_index.html]

Effects of topoclimatic complexity on the composition of woody plant communities — Supplementary Data 

# Effects of topoclimatic complexity on the composition of woody plant communities

## Supplementary Data

files

- Supplementary Data - zip file
