# Supplementary material for: Effects of topoclimatic complexity on the composition of woody plant communities
Source: AoB Plants. 2016 Aug 2;8:plw049. doi: 10.1093/aobpla/plw049 (PMC4972463; doi:10.1093/aobpla/plw049)
Supplement: Supplementary Data [file supp_plw049_suppl_data.zip › aobplants-16019-s01.docx]

**Supplementary Table 2.** Table. Mantel test results based on Pearson's product-moment correlation with 1e+05 permutations. These tests measure the correlation between the spatial distance between plots (SPATIAL) and the dissimilarity of the topoclimate principal components (PC1, PC2), and the correlation between SPATIAL and the vegetation community Bray-Curtis dissimilarity for both adult basal area (ADULT) and regeneration counts (REGEN).

|  |  | R^2^ | P value |
| --- | --- | --- | --- |
| PC1 | SPATIAL | 15.3 | 0.00242 |
| PC2 | SPATIAL | 10.0 | 0.04905 |
| ADULT | SPATIAL | 10.4 | 0.00741 |
| REGEN | SPATIAL | 16.1 | 0.00039 |
