# Supplementary figures and images for: Effects of topoclimatic complexity on the composition of woody plant communities
Source: AoB Plants. 2016 Aug 2;8:plw049. doi: 10.1093/aobpla/plw049 (PMC4972463; doi:10.1093/aobpla/plw049)

## Slide 1
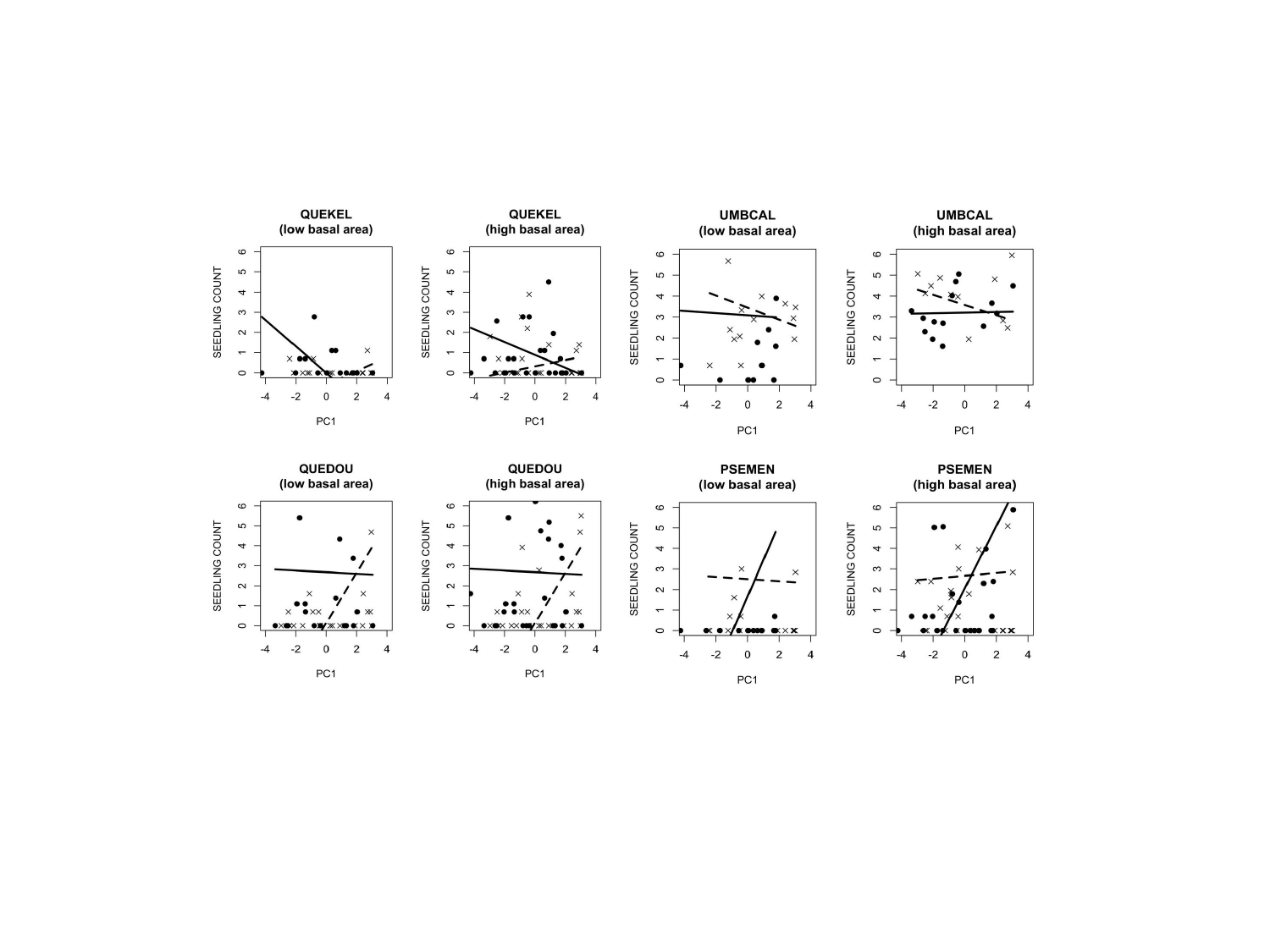

Supplement: Supplementary Data [file supp_plw049_suppl_data.zip › aobplants-16019-s03.pptx]
